# Characterization of a FGF19 Variant with Altered Receptor Specificity Revealed a Central Role for FGFR1c in the Regulation of Glucose Metabolism

**DOI:** 10.1371/journal.pone.0033603

**Published:** 2012-03-23

**Authors:** Hongfei Ge, Helene Baribault, Steven Vonderfecht, Bryan Lemon, Jennifer Weiszmann, Jonitha Gardner, Ki Jeong Lee, Jamila Gupte, Paramita Mookherjee, Minghan Wang, Jackie Sheng, Xinle Wu, Yang Li

**Affiliations:** 1 Amgen Inc., San Francisco, California, United States of America; 2 Amgen Inc., Thousand Oaks, California, United States of America; 3 Amgen Inc., Seattle, Washington, United States of America; University of Bremen, Germany

## Abstract

Diabetes and associated metabolic conditions have reached pandemic proportions worldwide, and there is a clear unmet medical need for new therapies that are both effective and safe. FGF19 and FGF21 are distinctive members of the FGF family that function as endocrine hormones. Both have potent effects on normalizing glucose, lipid, and energy homeostasis, and therefore, represent attractive potential next generation therapies for combating the growing epidemics of type 2 diabetes and obesity. The mechanism responsible for these impressive metabolic effects remains unknown. While both FGF19 and FGF21 can activate FGFRs 1c, 2c, and 3c in the presence of co-receptor βKlotho in vitro, which receptor is responsible for the metabolic activities observed in vivo remains unknown. Here we have generated a variant of FGF19, FGF19-7, that has altered receptor specificity with a strong bias toward FGFR1c. We show that FGF19-7 is equally efficacious as wild type FGF19 in regulating glucose, lipid, and energy metabolism in both diet-induced obesity and leptin-deficient mouse models. These results are the first direct demonstration of the central role of the βKlotho/FGFR1c receptor complex in glucose and lipid regulation, and also strongly suggest that activation of this receptor complex alone might be sufficient to achieve all the metabolic functions of endocrine FGF molecules.

## Introduction

The FGF19 subfamily of fibroblast growth factors (FGFs), consisting of FGF19, FGF21, and FGF23, is a novel group of endocrine factors that have been implicated in the regulation of many metabolic processes [1], [2], [3]. The subfamily members FGF19 and FGF21 share the ability to regulate glucose, lipid, and energy homeostasis. Both FGF19 and FGF21 transgenic mice are resistant to diet-induced obesity, have decreased adiposity and improved insulin sensitivity, glucose disposal, and plasma lipid profiles [4], [5]. Administration of recombinant FGF19 or FGF21 protein to diabetic mice resulted in the reduction of serum glucose and insulin levels, improved glucose tolerance, and reduced hepatosteatosis and body weight [6], [7], [8], [9], [10], [11]. In addition, FGF21 has also been shown to induce similar beneficial changes in rhesus monkeys [12]. These effects regarding correction of metabolic imbalances were potent and beneficial making FGF19 and FGF21 exciting new opportunities for exploring novel therapies to combat the growing diabetes and obesity epidemics.

The mechanisms leading to these impressive pharmacological changes are not well understood [13], [14], [15], [16]. One unique property of this subfamily is their distinct requirement for co-receptors. The paracrine-acting FGF molecules bind tightly to cell-associated heparan sulfate glycosaminoglycans and exert their actions by forming heparan-mediated high-affinity interactions with FGF receptors (FGFR) thereby activating receptor tyrosine kinases [17], [18], [19]. In contrast, FGF19 subfamily members have a weak affinity toward heparan sulfate of the pericellular space [20], [21], instead, they utilize single-transmembrane-containing Klotho proteins to facilitate their interactions with and activations of FGFRs. There are 2 related Klotho proteins: αKlotho and βKlotho. Both FGF19 and FGF21 utilize βKlotho for receptor interaction and activation [13], [14], [15], [16]. The FGFRs are encoded by 4 genes (FGFR1–FGFR4), while alternative splicing of FGFR1–3 further generates tissue-specific “b” and “c” isoforms [20], [21]. βKlotho interacts only with the “c” isoforms of FGFRs 1–3 and with FGFR4, therefore, restricting the potential receptor complexes that could be used by FGF19 and FGF21 as well as restricting the potential target tissues to those sites where both βKlotho and the appropriate FGFRs are expressed.

Both FGF19 and FGF21 activate FGFRs 1c, 2c, and 3c in a βKlotho-dependent manner in vitro [15], [22]. In addition, FGF19, but not FGF21, can also activate FGFR4 [10], [15], [22]. The only established link between a particular FGFR to physiological functions is the connection between FGFR4 activation to bile acid metabolism and hepatocyte mitogenesis. The involvement of FGFR4 activation to bile acid regulation was confirmed through the use of FGFR4 KO mice, and its involvement to hepatocyte mitogenesis was suggested through extensive studies with FGFR4 specific FGF19 molecules and various FGF19/21 chimeras with different FGFR specificity [23], [22]. Although it is believed that the metabolic activities of FGF19 and FGF21 are probably mediated through the activation of FGFRs1c, 2c, or 3c in the presence of βKlotho, whether it requires activation of all three or a subset of these receptors to achieve the glucose and metabolic effects of FGF19 and FGF21 is not understood. If one or a subset is sufficient to mediate these activities, which FGF receptor or receptors contribute to the observed glucose, lipid, and energy regulation by FGF19 and FGF21 also remains unknown.

Mitogenic activity has been observed in FGF19 transgenic mice, which developed hepatocellular carcinoma within 12 months and showed increased hepatocyte proliferation as early as 2 to 4 months of age [24]. The increased hepatocyte proliferation is also observed in wild type mice injected with recombinant FGF19 for 6 days [24]. We have recently shown that the activation of liver FGFR4 may be responsible for enhanced hepatocyte proliferation observed with a short 6 day treatment with FGF19 [22]. However, evidence connecting the proliferation observed in this short time frame to tumor formation in a chronic setting is still lacking.

In the present study, we generated a variant of FGF19, FGF19-7, that has altered receptor specificity with bias toward a βKlotho/FGFR1c receptor complex. This novel variant has proven to be a valuable tool in understanding which FGFR(s) are primarily responsible for the metabolic activities of FGF19 and FGF21.

## Results

### FGF19-7, a FGF19 variant with receptor specificity biased toward FGFR1c

**Figure 1:**
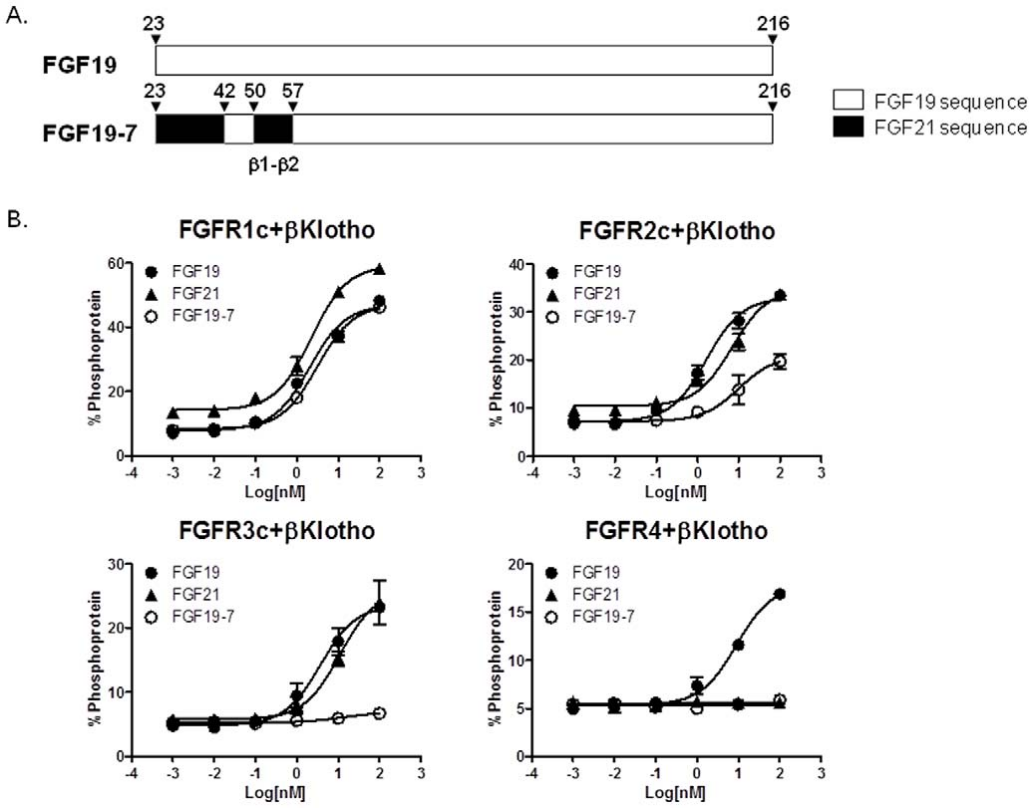
Receptor specificity for FGF19-7. (A) Schematic diagram showing FGF19 and FGF19-7. (B) L6 cells were co-transfected with expression vectors for FGFR1c, 2c, 3c, or 4 and βKlotho. Following overnight serum starvation, cells were stimulated with vehicle, recombinant FGF19, FGF21, or FGF19-7 for 15 min and snap frozen in liquid nitrogen. Cell lysates were prepared for MSD assay measuring ERK1/2 phosphorylation level.

Our previous studies suggested that the N-terminal region of endocrine FGF molecules is important for receptor specificity determination [25], [22], [26], [27]. Therefore, we generated and explored the activity of various N-terminal region chimeras between FGF19 and FGF21 to identify novel FGF molecules with altered receptor specificity. These investigations led to FGF19-7, a FGF19 variant that has significantly altered receptor specificity as compared to wild type (wt) FGF19 protein (Fig. 1).

FGF19-7 contains substitutions of FGF19 sequences from residues 23–42 and 50–57 with the corresponding sequences from FGF21 (Fig. 1A). The receptor specificity was studied in rat myoblast L6 cells, which express little endogenous FGFRs, and do not normally respond to FGF treatment. These cells were co-transfected with FGFR1c, 2c, 3c, or 4 and βKlotho. Signaling in response to FGF treatment was assessed by measuring phospho-ERK (p-ERK) levels using a semiquantitative MSD assay. Consistent with previous observations [10], [22], [27], while FGF19 was able to induce ERK phosphorylation with all four FGFRs (1c, 2c, 3c and 4) co-transfected with βKlotho in L6 cells, FGF21 activated only FGFRs 1c, 2c, and 3c with βKlotho but not FGFR4 (Fig. 1B). The receptor specificity profile of FGF19-7 was significantly different from both FGF19 and FGF21. While FGF19-7 fully activated FGFR1c/βKlotho, it had partial activity at FGFR2c/βKlotho and lacked activity at either FGFR3c or FGFR4 together with βKlotho (Fig. 1B). This pattern of specificity is summarized in Table 1. Therefore, FGF19-7 is biased toward activation of an FGFR1c/βKlotho receptor complex.

**Table 1:** Receptor specificity for FGF19, FGF21, and FGF19-7.

FGF molecules	Receptors			
	FGFR1c/βKlotho	FGFR2c/βKlotho	FGFR3c/βKlotho	FGFR4/βKlotho
**FGF19**	++	++	++	++
**FGF21**	++	++	++	−
**FGF19-7**	++	+	−	−

++ indicates strong activity.

+ indicates weak activity.

^−^indicates no activity.

### FGF19-7 showed similar metabolic efficacy to wt FGF19 in vitro and in vivo

**Figure 2:**
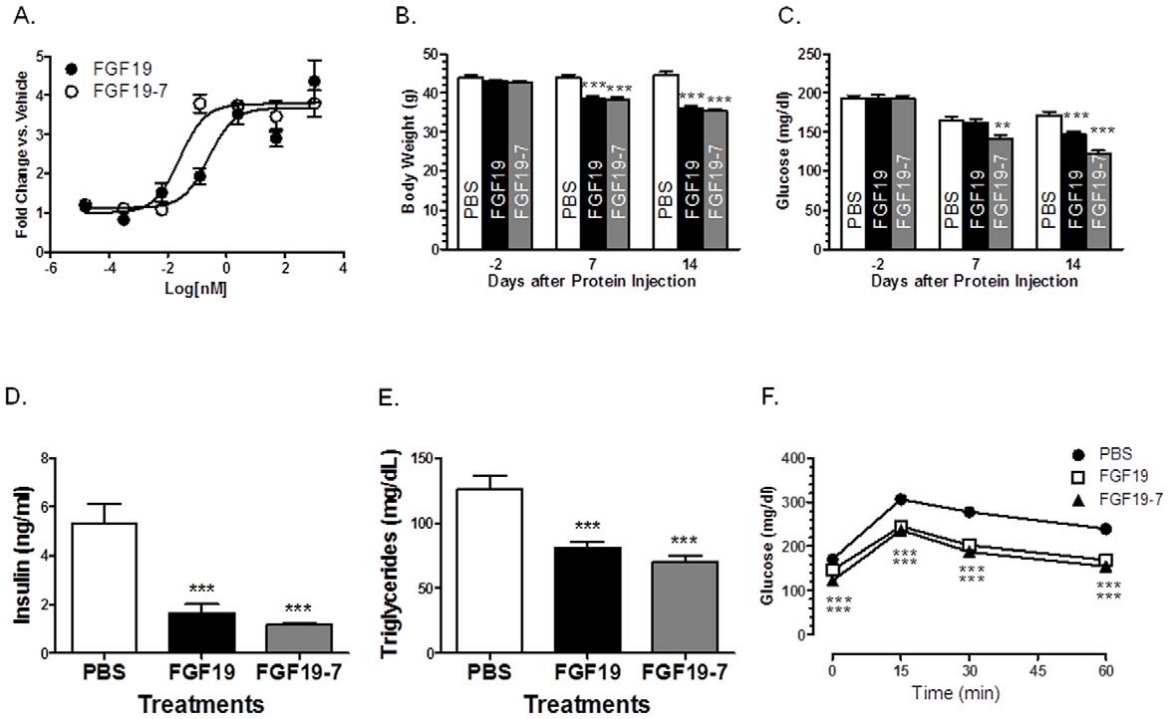
Effects of FGF19-7 on adipocyte glucose uptake and metabolic parameters in DIO mice. (A) Differentiated 3T3-L1 adipocytes were incubated for 72 h with FGF19 or FGF19-7 and assayed for glucose uptake. (B–F) Mice were injected with recombinant FGF19, FGF19-7 protein daily intraperitoneally at 1 mg/kg body weight or an equal volume of PBS control. Body weight (B) and fast glucose (4 hr fasting) (C) were measured 2 days before and 7 or 14 days after IP daily injection. After 14 days, an oral glucose tolerance test (OGTT) (F) was performed by administering 2 g/kg of glucose to each mouse and measuring serum glucose concentration at the indicated time points. Serum insulin (D) and triglyceride (E) levels were measured after 4 hr fasting. Values are means ± SEM of 12 mice/group. **P<0.01 and ***P<0.001, *t*-test.

We next tested FGF19-7 in various functional assays *in vitro* and *in vivo* to assess the effects of altered receptor specificity on the different functions of FGF19. We and others have previously shown that FGF19 can stimulate glucose uptake by adipocytes [15], [10], [22], [26], therefore, we first tested the effect of FGF19-7 in a glucose uptake assay using differentiated mouse 3T3-L1 adipocytes. As shown in Fig. 2A, FGF19-7 stimulated glucose uptake in adipocytes to a similar extent as FGF19 suggesting that the altered receptor specificity had no impact on this particular function of FGF19.

We further tested the ability of FGF19-7 to regulate glucose metabolism *in vivo* in both a diet-induced-obesity (DIO) murine model as well as leptin deficient *ob/ob* mice. Fourteen-week-old male B6D2F1 mice, fed a high-fat diet for 8 weeks, were divided into 3 groups (n = 12) based on initial body weight and glucose. Mice were then injected intraperitoneally (i.p.) with PBS, 1 mg/kg FGF19, or 1 mg/kg FGF19-7 daily for a period of 2 weeks. Compared to wild type FGF19, FGF19-7 showed equal reduction in body weight throughout the study (Fig. 2B), and equal reduction in plasma insulin (Fig. 2D) and triglyceride (Fig. 2E) levels at the termination of the study. The FGF19-7 group also showed a slightly better reduction in fasting glucose level compared to wild type FGF19. While significant reduction was achieved at 7 days post treatment with FGF19-7, no significant changes were observed in the wild type FGF19 treated group until day 14 (Fig. 2C). An oral glucose tolerance test (OGTT) was performed at the end of the 2-week treatment to assess the ability of the animals to dispose a glucose challenge. As shown in Fig. 2F, both FGF19-7 and FGF19 treatments significantly improved the responses of these animals to the oral glucose challenge (OGTT) to a similar extent.

A similar study was also carried out with *ob/ob* mice, whereby FGF19-7 showed equal efficacy to wild type FGF19 in lowering fasting plasma glucose levels and improving OGTT (data not shown). Similar or greater effects on the reduction of body weight and plasma insulin levels were also observed for the FGF19-7 group as compared with the wild type FGF19 group. Taken together, these results suggest that the ability of FGF19-7 to regulate glucose metabolism and to induce body weight reduction were unaffected despite the changes in receptor specificity.

### FGF19-7 lost the ability to induce mitogenesis and FGFR signaling in the liver

Besides the ability to regulate glucose metabolism, FGF19 has also been shown to induce hepatocyte proliferation mediated through the activation of liver expressed FGFR4 [24], [22], [26]. Since FGF19-7 lost the ability to activate FGFR4, we tested its effects on the induction of hepatocyte mitogenesis as well as activation of liver FGFR signaling.

**Figure 3:**
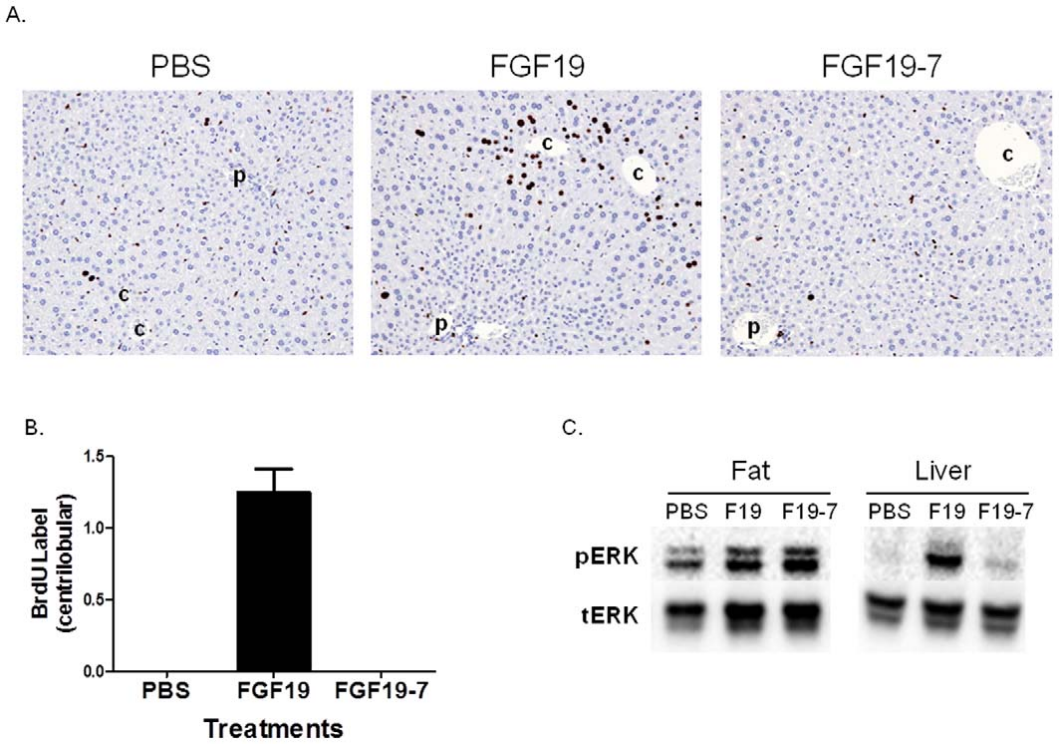
Effects of FGF19-7 on signaling and proliferation in liver in vivo. (A) BrdU immunostaining of livers from female FVB mice after BrdU infusion by osmotic minipump. Mice received daily injections of PBS (left), 2 mg/kg/day recombinant FGF19 (middle), or 2 mg/kg/day FGF19-7 (right) for 6 consecutive days beginning on day 2 of the study. Stained nuclei of hepatocytes in the liver from the FGF19 treated mouse are oriented around central veins (c) and away from portal veins (p). Hematoxylin counterstain. (B) Semiquantitative analysis of BrdU-positive hepatocytes from (A). The scores assigned to BrdU incorporation for these animals were based on a semiquantitative scale described in the Materials and Methods section. Solid bars represent group mean score with standard error (n = 8 for each group). (C) Liver and adipose tissue (epididymal fat) were excised from female FVB mice (four animals per group) treated with PBS, 10 mg/kg FGF19 (F-19) or FGF19-7 (F19-7) 15 min post injection. Tissue lysates were prepared and pooled for Western blot analysis using phosphorylated ERK1/2 (pERK) or total ERK1/2 (tERK) antibodies.

The effects of FGF19-7 on hepatocyte proliferation was tested using an *in vivo* BrdU labeling method similar to that described previously [24], [22], [26]. As shown in Fig. 3A and 3B, histopathological examination of liver sections from FGF19 treated animals showed increased BrdU-labeled hepatocytes concentrating in centrilobular regions of hepatic lobules, consistent with previously published observations [24], [22], [26]. In contrast, livers from FGF19-7 treated animals did not show increased BrdU labeling in hepatocytes in the pericentral regions, nor was increased BrdU incorporation noted in any other area of the liver. These results suggest that the FGF19 variant, FGF19-7, does not promote hepatocyte proliferation.

The FGFR signaling induced by FGF19 and FGF19-7 was directly measured in these animals as well. At the end of the 7 day study, liver and adipose tissue were collected 15 min following the final dose of FGF19 or FGF19-7 injection and ERK phosphorylation was measured by Western blot analysis (Fig. 3C). Consistent with the ability of both molecules to stimulate glucose uptake by 3T3-L1 adipocytes which mainly express FGFR1c (Fig. 2A), FGF19 and FGF19-7 stimulated ERK phosphorylation to a similar extent in adipose tissue in vivo (Fig. 3C left panel). In contrast, while wild type FGF19 induced robust signaling in the liver, no significant ERK phosphorylation was observed in livers from FGF19-7-treated mice. This is consistent with the notion that FGF19-7 lacks activity at FGFR4 which is the predominant receptor expressed in the liver (Fig. 3C right panel) and directly correlates with its inability to promote liver BrdU incorporation in vivo.

### One year study with FGF19-7 using AAV gene delivery

The ability of FGF19 to induce hepatocyte proliferation observed in the BrdU incorporation studies has been suggested as the cause for the eventual development of hepatocellular carcinomas in FGF19 transgenic mice [24]. Because FGF19-7 does not induce hepatocyte proliferation, it could be used as a tool to test the link between BrdU incorporation observed by acute FGF19 treatment in a 1-week study to the long term effect of tumor development.

The observation that FGF19 induced liver tumors was made with a transgenic model, thus potential developmental contributions of the transgene to tumor formation could not be ruled out. Stable long term expression of up to 1 year has been observed with the AAV gene delivery method ([28], JS unpublished observations). We decided to assess the long term effect with respect to tumor formation of sustained expression of FGF19 and FGF19-7 using AAV as a gene delivery vehicle. Additionally, in order to obtain information on the metabolic benefits of FGF19 and FGF19-7 in an adult on-set model of obesity, B6D2F1/J male mice were first put on high fat diet at 3–4 weeks old prior to AAV virus injection. The study was carried out for 1 year with periodic measurements of body weight and glucose. At the conclusion of the study, body weight, liver weight, plasma glucose, OGTT, TG, insulin, and FGF19 levels were measured. In addition, the proliferation state of hepatocytes was assessed by implantation of a minipump containing BrdU one week prior to termination by a procedure similar to the study shown in Fig. 3A and histopathologic evaluation of liver sections was performed.

**Figure 4:**
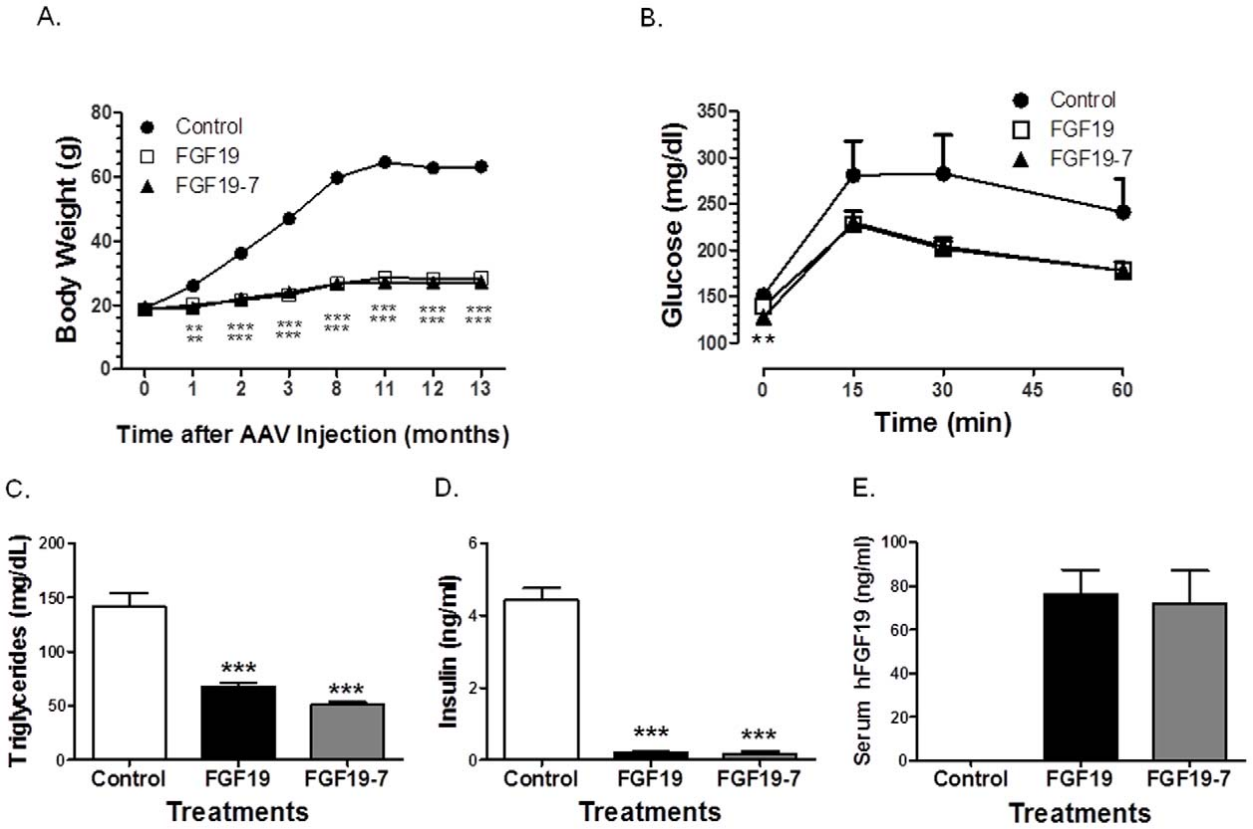
Metabolic parameters in DIO mice after 1 year treatment with FGF19-7. (A) Body weight. Mice receiving FGF19 and FGF19-7 remained leaner than control 13 months after AAV injection. Eleven months after AAV administration, oral glucose tolerance test (OGTT) (B) was performed by injecting mice with 2 g/kg of glucose and measuring serum glucose concentration at indicated time points. Triglyceride (C), insulin (D) and serum human FGF19 levels (E) were measured after 4 hr fasting prior to termination of the study. Values are means ± SEM of 10–15 mice/group. **P<0.01 and ***P<0.001, *t*-test.

During the course of the 1 year study, mice injected with AAV expressing FGF19-7 had reduced body weight gain similar to the group receiving AAV expressing wild type FGF19 (Fig. 4A). The fasting glucose levels were not significantly different between all groups (data not shown), however, mice receiving AAV virus expressing FGF19 and FGF19-7 significantly improved their response to an oral glucose challenge (Fig. 4B). In addition, at the termination of the study, both FGF19 and FGF19-7 groups had significantly lowered plasma TG and insulin levels compared to control, consistent with results from the short term studies (Fig. 2) and previously published effects of FGF19 on metabolism. These observations further verified that selective activation of an FGFR1c/βKlotho receptor complex was sufficient to elicit the metabolic regulation observed with wild type FGF19.

**Figure 5:**
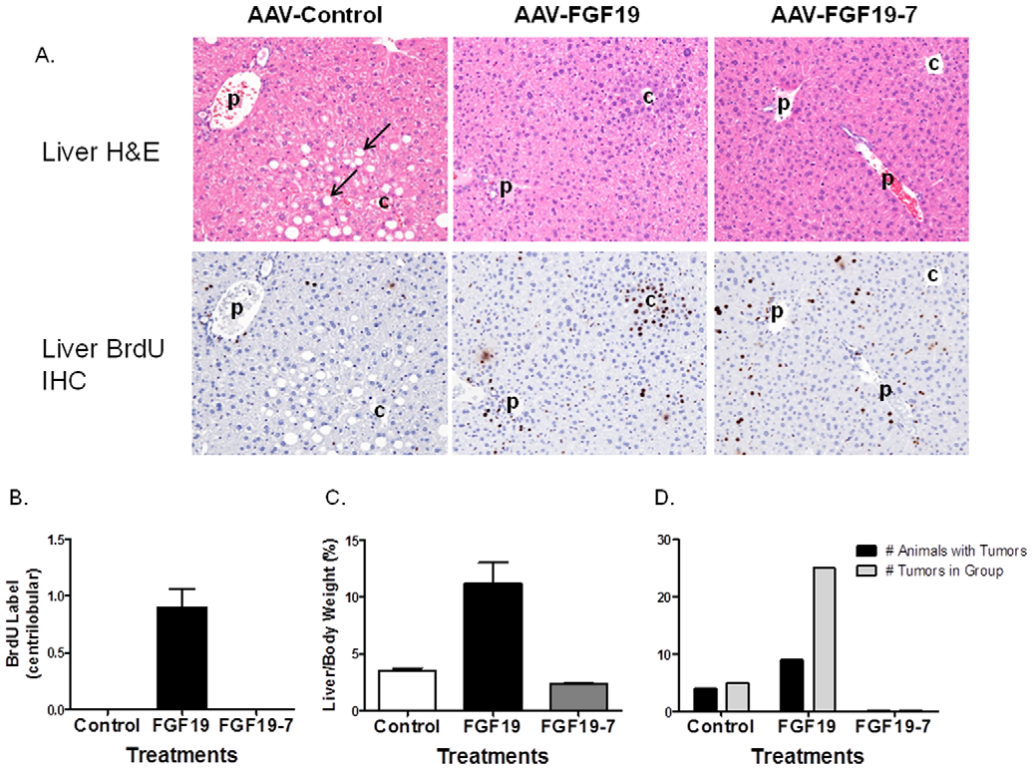
Liver phenotype of DIO mice after 1 year treatment with FGF19 and FGF19-7. (A) Liver sections from mice inoculated 1 year prior with AAV-Control (left panel), AAV-FGF19 (middle panel), or AAV-FGF19-7 (right panel) virus and stained with H&E (top panel) or by an immunohistochemical method to visualize BrdU incorporation as a marker for DNA synthesis and mitosis (bottom panel). The sections stained for BrdU incorporation were taken from an area near to the paired section stained with H&E and show the same portal (p) and central (c) veins. Centrilobular to midzonal hepatocytes in the H&E-stained section of liver from the mouse inoculated with AAV-Control show cytoplasmic vacuolation (arrows) that likely reflects fat accumulation due to consumption of a high fat diet. Mice inoculated with AAV-FGF19 or AAV-FGF19-7 were resistant to this effect of the high fat diet. Hematoxylin counterstain (bottom panel); all photographs 100×. (B) Semiquantitative analysis of BrdU-positive hepatocytes from (A bottom panel). (C) Liver weight comparison between the different groups. (D) Quantitative counts of number of tumors present in the different groups at the end of the 1 year study.

The morphology of the livers from the 3 treatment groups showed dramatic differences. Livers from control AAV virus treated mice were pale consistent with accumulation of fat in hepatocytes from high fat feeding. This was visualized as cytoplasmic vacuolation (arrows, Fig. 5A top panel) in the H&E-stained liver sections from mice inoculated with AAV-Control virus. While animals receiving AAV-FGF19 virus lacked signs of lipid accumulation in liver (Fig. 5A, top panel), gross morphological changes with multiple nodules or masses were observed in their livers consistent with the formation of liver tumors. AAV-FGF19 virus treated animals also showed a dramatic increase in liver weight compared to the other two groups (Fig. 5C). In contrast, the livers from AAV-FGF19-7 virus treated animals had normal gross morphology and no fat accumulation was observed (Fig. 5A).

Sections of H&E stained livers from these mice (Fig. 5A, top panel) were evaluated for the presence of tumors and lesions considered preneoplastic such as foci of hepatocellular alteration and intravenous protrusion of hepatocytes. In addition, BrdU staining was carried out to evaluate hepatocellular proliferation (Fig. 5A, bottom panel). The sections stained for BrdU incorporation were taken from an area near to the paired section stained with H&E and the photographs show the same portal (p) and central (c) veins. Benign or malignant tumors were present in 4 of the 11 AAV-control mice and a focus of cellular alteration was present in one additional mouse from this group. This could be expected because C57BL/6 mice are susceptible to the development of liver tumors when fed a high-fat diet [29], [30]. Nine of the 10 mice inoculated with AAV-FGF19 displayed benign hepatic tumors (hepatocellular adenoma; 6 mice), malignant hepatic tumors (hepatocellular carcinoma; 7 mice) or both (4 mice). Preneoplastic hepatocellular lesions (foci of hepatocellular alteration, intravenous protrusion of hepatocytes or both) were noted in 8 of these 10 mice including the one mouse in this group that lacked a hepatocellular tumor (Fig. 5D). Examination of non-tumor bearing portions of the liver showed that hepatocytes around central veins in the H&E-stained sections of livers from mice inoculated with AAV-FGF19 were crowded, slightly smaller, and had increased cytoplasmic basophilia when compared with hepatocytes in the same area of livers from mice inoculated with AAV-FGF19-7 (Fig. 5A). Remarkably, none of the livers from the 11 mice inoculated with AAV-FGF19-7 exhibited tumors or preneoplastic lesions.

Immunohistochemistry for BrdU incorporation showed that mice inoculated with AAV-FGF19 incorporated BrdU tightly restricted to hepatocytes around the central vein similar to observations in mice acutely treated with recombinant FGF19 protein for 6 days (Fig. 5A bottom panel). This was not seen in mice that received control AAV or AAV-FGF19-7 constructs. Mice inoculated with AAV-FGF19-7 exhibited equivocally to minimally increased numbers of BrdU-labeled hepatocytes compared to the AAV control group, but these lacked orientation to central veins or other portions of the hepatic lobules. However, given that the metabolic homeostasis and, in particular, the state of the liver between these two groups are dramatically different, i.e. the AAV control group exhibited steatosis not seen in the FGF19-7 group, it was difficult to conclude whether these differences could contribute to background BrdU signals. More importantly, the pericentral BrdU staining seen in the wild type FGF19 group was absent in the FGF19-7 group and no tumor formation was observed in the latter indicating that the FGF19 variant had lost the ability to induce liver tumor formation.

## Discussion

FGF19 and FGF21 are unique members of the FGF family and have been shown as novel hormones that can regulate glucose, lipid, and energy metabolism. Their potent effects on improving glucose disposal, insulin sensitivity, plasma lipid parameters, and on inducing body weight loss are very similar between these two endocrine FGFs and have been reported in multiple models of diabetes and obesity in rodents and non-human primates. However, the underlying mechanisms leading to these metabolic benefits are not well understood.

Not only do FGF19 and FGF21 share similar in vivo pharmacology with respect to glucose regulation, they also share many similarities in receptor activities. Both FGF19 and FGF21 have reduced or no affinity to heparan sulfate and use a single pass transmembrane protein βKlotho to interact with and to activate FGFRs [13], [15], [14]. In cell based assays in vitro, both FGF19 and FGF21 can activate FGFRs 1c, 2c, and 3c when complexed with βKlotho. The only difference in receptor utilization reported for these two FGFs is that FGF19, but not FGF21, can activate FGFR4 [31]. Therefore, it was known that both FGF19 and FGF21 can activate multiple FGF receptor complexes in vitro; however, their metabolic activity, such as regulation of glucose metabolism, had not been clearly ascribed to a specific FGFR/βKlotho complex prior to this study.

To identify the receptor or receptors responsible for the metabolic activities of FGF19 and FGF21, we took the approach to generate FGF19 or FGF21 variants that selectively activate one or a subset of the FGFRs. We previously demonstrated that such an approach is feasible with the generation of an FGF19 variant, FGF19dCTD, which is a specific activator of FGFR4 [10]. In the diabetic ob/ob mouse model, we showed that selective activation of FGFR4 was not sufficient to improve glucose metabolism suggesting that other receptors must be important for the regulation of glucose homeostasis [10]. Because the primary difference between FGF19 and FGF21 is the ability of FGF19 to activate FGFR4, yet both have similar effects on glucose metabolism, these results suggest that the metabolic effects of FGF19 and FGF21 are likely mediated through a similar mechanism and through receptors other than FGFR4. Therefore, the understanding of FGF19 induced metabolic effects such as glucose regulation could be translated to FGF21 and vice versa.

Because we recently showed that the N-terminal regions of FGF19 and FGF21 are important for receptor specificity determination [31], we explored various N-terminal mutations to generate an FGF variant that can selectively activate FGFR1c, 2c, or 3c, the 3 receptors commonly activated by FGF19 and FGF21, in complex with βKlotho. From many different variants we have tested, FGF19-7 was identified to have a receptor specificity biased toward the FGFR1c/βKlotho complex. Despite receptor specificity significantly altered from FGF19, FGF19-7 was equal to or better than wild type FGF19 at increasing glucose uptake into adipocytes in vitro and at reducing body weight, plasma insulin, glucose, and TG levels, and at improving glucose disposal in both diet induced obesity and ob/ob mice models. These results provide the first strong in vivo evidence that the FGFR1c/βKlotho receptor complex may be the main, if not the sole receptor complex that mediates the metabolic effects of FGF19 and by extension, FGF21 as well. Consistent with the expression of FGFR1c being predominately in adipocytes among the βKlotho expressing cells, FGF19-7 was only able to activate FGFR signaling in fat but not liver (Fig. 3C). This suggests that fat maybe the primary target tissue for the metabolic functions of FGF19 and FGF21.

In addition to its ability to regulate glucose metabolism, FGF19 was reported to induce hepatocyte proliferation. This conclusion was based on the observation that BrdU incorporation increased in the liver of animals injected with FGF19 protein for 6 days, especially in hepatocytes adjacent to central veins [24]. In a chronic setting, hepatocellular carcinoma (HCC) formation was observed in 8–10 month old FGF19 transgenic mice [24]. It was, therefore, suggested that the induction of hepatocyte proliferation observed in the relatively short BrdU study was responsible for the formation of HCC in the FGF19 transgenic animals [24]. We previously showed that the hepatocyte proliferation observed in the short term studies is due to activation of liver FGFR4 receptor by FGF19 [22], [26], however, no direct link between short-term BrdU labeling and long-term HCC formation was previously established.

Because FGF19-7 does not activate FGFR4 (Fig. 1B), its effect on hepatocyte proliferation and on HCC formation were studied. In contrast to FGF19 treated animals, mice receiving recombinant FGF19-7 protein for 7 days did not show increased BrdU labeled hepatocytes in the pericentral region or in any other areas of liver (Fig. 3). The effect of FGF19 versus FGF19-7 on liver tumor formation was also studied utilizing AAV gene delivery method to introduce these proteins into mice starting at 4-weeks age. Sustained expression of FGF19 or FGF19-7 was observed in our study after 1 year (Fig. 4E). The animals were also subjected to a high fat diet during the 1 year treatment, such that their long term effect on metabolism could also be monitored. At the end of the long-term study, similar to the 2 week study, FGF19-7 showed effects equal to or better than FGF19 on reducing the body weight gain and elevated plasma insulin and TG levels caused by the high-fat diet. Improved glucose disposal was also observed (Fig. 4). However, a major difference between the FGF19 and FGF19-7 groups was that the FGF19 group developed liver tumors, while no liver tumors were observed in the AAV-FGF19-7 group (Fig. 5). Our method, using AAV-FGF19-treated mice, confirmed that chronic exposure to FGF19 as observed in the FGF19 transgenic mice causes formation of hepatocellular tumors. In addition, the lack of liver tumors in the FGF19-7 group further establishes the connection of FGFR4 activation and enhanced BrdU labeling in short-term studies to liver tumor formation in long-term studies. Interestingly, liver tumors were also observed in the AAV control group on a high fat diet. This suggests that FGF19-7, either through the beneficial metabolic effects or directly, suppressed or protected these animals from the development of hepatocellular tumors.

Taken together, these results not only confirmed that the activation of the FGFR1c/βKlotho receptor complex is not expected to induce hepatocyte proliferation and HCC formation, but further established its central role in mediating the metabolic activities of FGF19 and FGF21.

## Materials and Methods

### Cell culture and transfections

L6 cells (obtained from American Type Culture Collection) were maintained in Dulbecco's modified Eagle's medium supplemented with 10% fetal bovine serum and penicillin/streptomycin. Cells were transfected with expression vectors harboring FGF19 and variants by using the Lipofectamine 2000 transfection reagent (Invitrogen) according to the manufacturer's protocol.

### MSD assay and Western analysis for FGF signaling

Briefly, L6 cells were plated, transfected with FGF receptors and treated with various FGF molecules as previously described [10]. Cells were collected 15 min after treatment, snap frozen in liquid nitrogen, lysed in the lysis buffer, and the total and phosphorylated ERK were measured by using an MSD whole cell lysate Phospho-ERK1/2 kit (Meso Scale Discovery) according to the manufacturer's instructions. All experiments were run in duplicates.

### In vivo hepatocyte BrdU labeling

In vivo BrdU labeling studies were carried out as previously described [22]. On day 1 of the short-term labeling study, an osmotic minipump (ALZET®, model 1007D) containing 5-bromo-2′-deoxyuridine (BrdU; Sigma Chemical Co., St. Louis, MO) (16 mg/mL) was implanted subcutaneously. Samples of liver and duodenum were collected from each mouse on day 7 after the minipump implantation and placed in 10% neutral-buffered formalin in preparation for paraffin-embedding, sectioning, and light microscopic evaluation. The section of duodenum was collected as a positive control for BrdU release from the minipump and for the BrdU immunohistochemical staining procedure since the epithelium of this tissue has a rapid turnover rate and should show complete labeling for BrdU incorporation after 7 days of exposure to BrdU. Sections of all collected tissues were stained by an immunohistochemical method to visualize BrdU incorporation as a marker of mitotic activity. Tissue sections were examined at random by routine light microscopy without knowledge of treatment group. The number of hepatocyte nuclei stained for BrdU incorporation was assigned a score on a semiquantitative scale as follows: 0 =  no increase above expected levels in vehicle-treated (control) mice and ± =  equivocal, 1 =  minimal, 2 =  mild, 3 =  moderate, and 4 =  marked increase above control levels. The localization (centrilobular or diffusely scattered through hepatic lobules) of the hepatocytes stained for BrdU incorporation was also recorded. Only hepatocyte nuclei (large, round nuclei clearly within hepatocytes) were considered for semiquantitative scoring of BrdU labeling. Nuclei of other cells types (e.g., bile duct epithelium, Kupffer cells, endothelial cells, and infiltrating leukocytes) were sometimes labeled with BrdU; however, these cells and their nuclei are morphologically distinct from hepatocytes and hepatocyte nuclei and have different anatomic localizations. These nuclei were not considered for the scoring of BrdU labeling in hepatocytes. Labeling with BrdU in the long-term AAV study was done in the same manner. The BrdU-containing minipump was implanted 7 days before collection of tissues.

### Mice and treatment

All animal experiments were approved by the Institutional Animal Care and Use Committee of Amgen. Mice were housed in an air-conditioned room at 22±2°C with a 12 h light: 12 h darkness cycle (0600–1800 h). Male B6D2F1 mice were purchased from Charles River. Two days before AAV injection, cohorts of 4-week-old mice (n = 15) were sorted by body weight and fasting glucose levels. Indicated adeno-associated virus (AAV) in 200 µl of PBS was administrated via the tail vein with a U-100 insulin syringe. The dose was 8×10^12^ virus particles per mouse. After AAV administration, all the mice were put on BioServe 1850 high-fat diet (60 kcal % fats) through the end of study.

Fourteen-week-old male B6D2F1 mice fed with Research Diets D12492 (60 kcal % fat) for 8 weeks were purchased from Jackson Laboratory. Two days before protein injection, mice were divided into 3 groups (n = 12) based on body weight and glucose. Starting from day0, mice were intraperitoneally-injected daily with recombinant FGF19 or FGF19-7 protein in 0.2 ml PBS (each at 1 mg/kg body weight) or same volume of PBS control. OGTT was done on day7 and 14, following 4 hr fasting. Terminal bleeds were taken at day15, following 6 hr fasting.

### Glucose tolerance tests and plasma insulin, triglyceride and FGF19 measurement

Mice were fasted for 4 hr beginning at 6 am on the day of the experiment. Blood samples obtained from the tail vein were used for insulin, triglyceride and FGF19 measurements. Following administration of glucose (2 g per kg oral gavage), glucose levels were measured immediately predose, 15, 30, and 60 min after glucose injection by using Accu-chek Aviva blood glucose meter (Roche Diagnostic). Plasma insulin content was determined by using Insulin (mouse) ultra-sensitive EIA kit (ALPCO Diagnostics, 80-INSMSU-E10). Plasma triglyceride was measured by using Serum Triglyceride Determination Kit (Sigma, TR0100). Plasma FGF19 level was determined by an in house ELISA assay using polyclonal Anti-human FGF19 antibody and Biotinylated anti-human FGF19 antibody (R&D systems, Catalog # AF969 and # BAF969).

### Western-blot analysis of the FGF signaling pathway

Liver and adipose tissue were collected 15 min after injection of indicated proteins and snap frozen in liquid nitrogen. After homogenization in lysis buffer, liver and fat samples were separated on 4–15% polyacrylamide gels (Bio-Rad) and transferred to nitrocellulose membranes (0.45 µM, Bio-Rad). Membranes were blocked (5% non-fat dry milk in 0.05% Tween-PBS) and incubated with a polyclonal anti-rabbit/phospho-ERK antibody (1∶2000, Cell Signaling #9101). Bound antibody was detected by peroxidase-conjugated secondary antibody and visualized using SuperSignal West Pico Chemiluminescent Substrate (Pierce Biotechnology). The same membranes were washed and used for total ERK analysis by using anti-rabbit/ERK antibody (1∶2000, Cell Signaling #9102).
